# Magnetic resonance imaging with gradient sound respiration guide

**DOI:** 10.1371/journal.pone.0254758

**Published:** 2021-07-19

**Authors:** Naoharu Kobayashi

**Affiliations:** Center for Magnetic Resonance Research, Department of Radiology, University of Minnesota, Minneapolis, MN, United States of America; McLean Hospital, UNITED STATES

## Abstract

Respiratory motion management is crucial for high-resolution MRI of the heart, lung, liver and kidney. In this article, respiration guide using acoustic sound generated by pulsed gradient waveforms was introduced in the pulmonary ultrashort echo time (UTE) sequence and validated by comparing with retrospective respiratory gating techniques. The validated sound-guided respiration was implemented in non-contrast enhanced renal angiography. In the sound-guided respiration, breathe−in and–out instruction sounds were generated with sinusoidal gradient waveforms with two different frequencies (602 and 321 Hz). Performance of the sound-guided respiration was evaluated by measuring sharpness of the lung-liver interface with a 10–90% rise distance, *w*_10-90_, and compared with three respiratory motion managements in a free-breathing UTE scan: without respiratory gating (w/o gating), 0-dimensional k-space navigator (k-point navigator), and image-based self-gating (Img-SG). The sound-guided respiration was implemented in stack-of-stars balanced steady-state free precession with inversion recovery preparation for renal angiography. No subjects reported any discomfort or inconvenience with the sound-guided respiration in pulmonary or renal MRI scans. The lung-liver interface of the UTE images for sound-guided respiration (*w*_10-90_ = 6.99 ± 2.90 mm), k-point navigator (8.51 ± 2.71 mm), and Img-SG (7.01 ± 2.06 mm) was significantly sharper than that for w/o gating (17.13 ± 2.91 mm; *p* < 0.0001 for all of sound-guided respiration, k-point navigator and Img-SG). Sharpness of the lung-liver interface was comparable between sound-guided respiration and Img-SG (*p* = 0.99), but sound-guided respiration achieved better visualization of pulmonary vasculature. Renal angiography with the sound-guided respiration clearly delineated renal, segmental and interlobar arteries. In conclusion, the gradient sound guided respiration can facilitate a consistent diaphragm position in every breath and achieve performance of respiratory motion management comparable to image-based self-gating.

## Introduction

Respiratory motion management is crucial for high-resolution magnetic resonance imaging (MRI) of the heart, lung, liver and kidney. The diaphragm during relaxed respiration typically moves 1–3 cm mainly in the superior-inferior direction [1–4]. Such diaphragmatic motion during MRI scans is usually monitored with a respiration bellows for gated MRI data acquisition. However, physiological monitoring using a respiration bellows is often poorly coupled with the diaphragmatic motion, which may result in inaccurate respiratory motion estimation [5–7]. Breath-holding is another frequently used strategy to compensate respiratory motion during MRI scans. However, breath-holding limits the scan time to typically 10–20 seconds and can be challenging for patients with reduced lung function. Use of multiple breath-holds can mitigate the scan time limitation, but inconsistent diaphragm position in each breath-hold often results in residual motion artifacts [8].

Navigator acquisition such as a spin-echo signal of an intersection of excitation and refocusing slices [9, 10] and pencil-beam excitation with a 2D selective radiofrequency (RF) pulse [11, 12] are also widely used to track the diaphragmatic motion during free-breathing and to prospectively trigger MRI data acquisitions. Triggering with navigators does not require special hardware for motion tracking, which can simplify procedure in MR exams. However, while diaphragm position tracking with navigators can significantly reduce respiratory motion artifacts, it may result in lengthy scan time especially when the respiration pattern is unstable and/or a narrow acceptance window is employed. To improve the stability of the diaphragm position, a respiratory biofeedback system was previously introduced, where the diaphragm position measured by spin-echo navigator and a target diaphragm position at end-exhalation were presented on a projection screen in real time during MRI scans [13]. The subject’s respiration was guided to match the diaphragm position to the target by playing a “game” displayed on the projection screen.

Other types of navigators have been developed over the decades for prospective and retrospective respiratory gating. k-space center navigator (also implemented as DC/FID navigator) is a well-established navigation technique for respiratory and cardiac motion tracking [5, 14–18]. The k-space center signal intensity represents an integral of signals in the excited volume (i.e., 0-dimensional (0D) projection), fluctuation of which mostly reflects motion-related changes and blood inflow in the imaging volume. In previous studies, different information of the k-space center signals such as magnitude, phase, real and imaginary was used as a gating signal. Respiratory and/or cardiac motion was successfully extracted from the k-space center signals. The k-space center navigator is widely used with the 3D radial ultrashort echo time (UTE) sequence, since the k-space center is inherently sampled in every pulse repetition time (TR) period such that separate navigator acquisitions are not required. Previous works have demonstrated that pulmonary UTE MRI using retrospective respiratory gating with the k-space center signal can successfully compensate respiratory motion during free-breathing [19, 20].

To capture more accurate respiratory motion information, image-based self-gating has been introduced, where 2D or 3D images were acquired with a subsecond temporal resolution for tracking respiratory motion during MRI scans [15, 21–25]. In image-based self-gating, high temporal resolution imaging is achieved by fast image-based navigator acquisition with spiral k-space sampling [22, 23] or balanced steady-state free precession (bSSFP) readout [24, 25]. Radial k-space sampling is also frequently used in image-based self-gating, since it does not require separate navigator acquisitions [15, 21]. In radial MRI reconstruction, sliding-window reconstruction can be easily applied to improve nominal spatial/temporal resolution. Recently, image-based self-gating has been implemented in pulmonary 3D radial UTE MRI to compensate respiratory motion during free breathing [7, 26, 27] and demonstrated that image-based self-gating works better than the k-space center navigator.

In this article, a respiratory motion compensation method that guides subjects’ breathing during MRI scans using acoustic noise of the gradient coil set is introduced. Vibration of the gradient coil windings caused by switching of pulsed gradients in strong static magnetic field generates unpleasant acoustic noises during MRI scans. To reduce such acoustic noises, gradient waveforms were previously designed by avoiding acoustic resonant harmonics of the gradient system [28–30]. More recently, musical sound was played by designing tailored pulsed gradient waveforms [31]. In this work, two characteristic sounds were generated for breathing instructions using pulsed gradient waveforms: one for inhalation and the other for exhalation. The gradient sound respiration guide synchronized with the MRI pulse sequence facilitates study subjects to breathe with consistent respiration rate and pattern during MRI scans, which can guide similar diaphragm position in every breath.

The sound-guided respiration was implemented in the pulmonary UTE MRI sequence. Performance of the sound-guided respiration was tested by comparing with free-breathing UTE MRI images reconstructed with two retrospective respiratory gating methods: 0D navigator referred to as k-space point navigator herein and image-based self-gating. Sharpness of the lung-liver interface at the top flat region of the right hemidiaphragm was quantified. To evaluate the stronger blood inflow effects in the sound-guided respiration UTE MRI, signal-to-noise ratio (SNR) in the lung tissues was compared for the three respiratory motion managements. In pulmonary UTE images, since vasculature was clearly visualized with strong contrasts between blood and lung tissues, visualization of pulmonary vasculature was compared in maximum intensity projection (MIP) of the pulmonary UTE images. Finally, the sound-guided respiration was implemented in non-contrast enhanced renal angiography with inversion recovery preparation.

## Materials and methods

MRI studies were conducted under a protocol approved by Institutional Review Board in the University of Minnesota with the protocol number 1212M24883. Written informed consent was obtained from all healthy volunteer subjects before MRI exam. Eight and seven subjects were recruited in pulmonary imaging (age 32.5 ± 14.7 years, 5 male and 3 female) and renal imaging (age 28.3 ± 9.3 years, 4 male and 3 female), respectively. MRI scans were performed with a 3 T MRI system (Prisma, Siemens Medical Solutions, Erlangen, Germany) and a combination of body matrix and spine coils for signal reception.

### Respiration guide with gradient acoustic sound

To guide breathing of the subjects during MRI scans, two distinctive sounds were generated by characteristic pulsed gradient waveforms during MRI scans. The respiration guide sound was generated by sinusoidal gradient waveforms with frequencies of 602 and 321 Hz for inhalation and exhalation guide, respectively. The sinusoidal gradient waveforms were 400 ms long and applied in all three channels of the gradient coil set. The two frequencies and duration were empirically selected to generate clearly distinguishable sounds. Respiration was guided to 6 sec/breath (10 breaths/min), where 1.8 and 4.2 sec was assigned for inhalation and exhalation, respectively. MRI data acquisition was performed during the latter half of the exhalation period. Accordingly, during MRI scans with the respiration guide, the subjects heard three types of sounds in every 6-sec period: 1) high pitch breathe-in instruction, 2) low pitch breathe-out instruction, and 3) sound from MRI data acquisition.

After obtaining informed consent, the sound-guided respiration was explained to the study subjects using a sound recording of the respiration guide before moving to the MRI room (S1 Audio). The subjects were instructed to breathe with a similar/consistent breathing depth in every breath in sound-guided MRI scans. During MRI data acquisition in the exhalation period, the subjects were instructed to stay relaxed after breathing out (i.e., no intentional breath-hold). At the beginning of each MRI exam, we ran one short practice scan with duration of 1:30 (15 breaths), during which the subjects practiced the sound-guided respiration and found comfortable breathing depth and pattern in the MRI scanner.

### Pulmonary imaging

**Fig 1:**
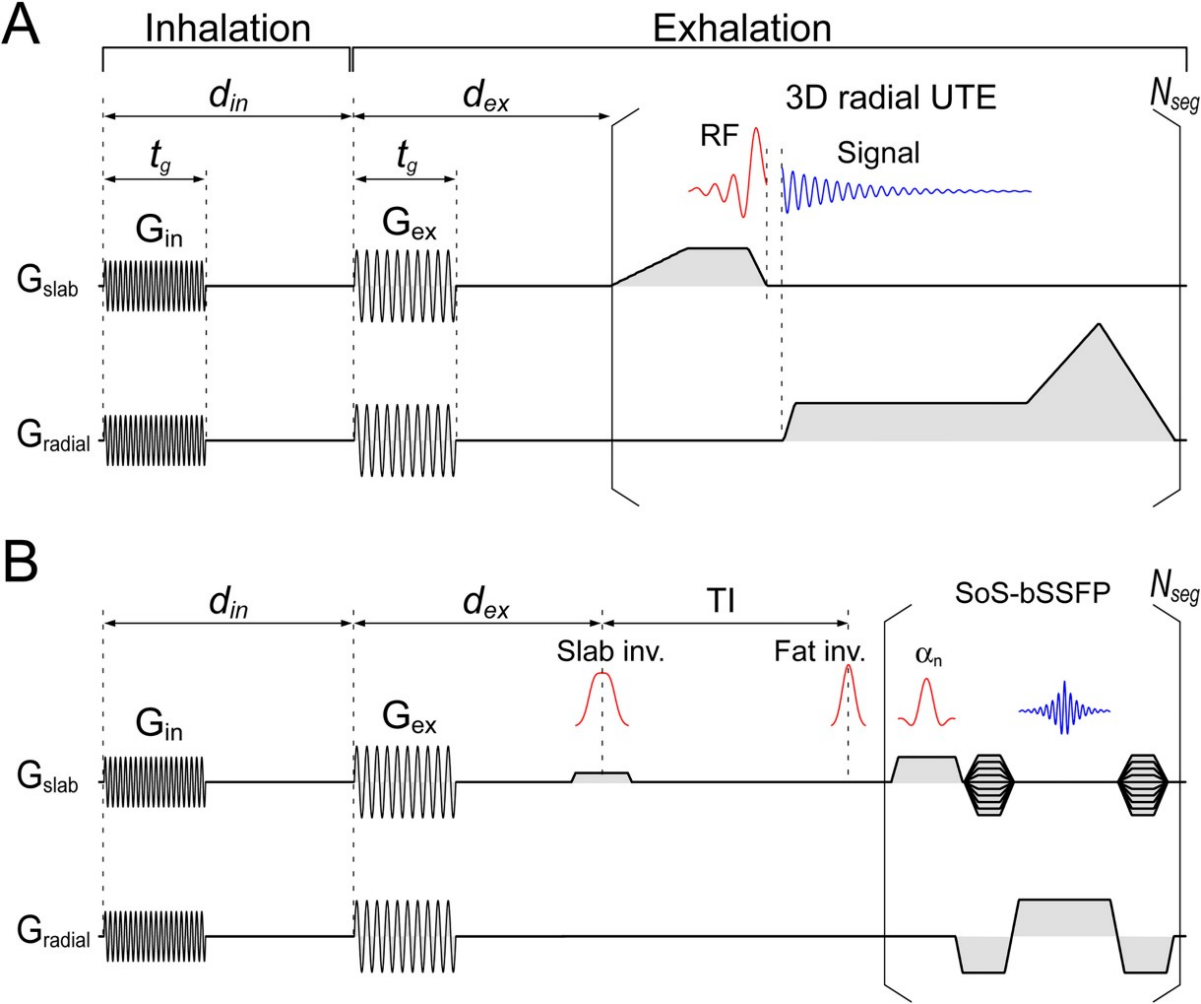
Sequence diagram of sequences with gradient-sound respiration guide. A) UTE sequence with sound-guided respiration. Respiration instruction sounds were generated by sinusoidal gradient waveforms, G_in_ and G_ex_, with a duration *t*_*g*_ = 400 ms; frequency of the gradient sound was set to 602 and 321 Hz for inhalation and exhalation instruction, respectively. Respiration was guided with 6 sec/breath, where 1.8 (= *d*_*in*_) and 4.2 sec was assigned for inhalation and exhalation, respectively. Segmented UTE scan started at *d*_*ex*_ = 1.6 sec after the beginning of the exhalation instruction. B) Stack-of-stars balanced steady-state free precession (SoS-bSSFP) sequence with sound-guided respiration. The gradient sound guide was identical to that in the pulmonary UTE scan (1.8 and 4.2 sec for inhalation and exhalation, respectively). Slab-selective spin inversion for background signal suppression was applied at *d*_*ex*_ = 800 ms after the onset of the exhalation instruction. Segmented SoS-bSSFP data acquisition started following an inversion delay of TI = 1300 ms and spectral-selective inversion for fat suppression.

Performance of the sound-guided respiration was investigated with pulmonary UTE MRI. UTE acquisitions were inserted after 1.6 sec from the onset of the exhalation instruction (Fig 1A). UTE data acquisition was performed during 46 breaths with *N*_*seg*_ = 894 radial views per breath, which resulted in 41,124 radial views and scan time 4:36. 3D k-space was sparsely sampled every 894 views (every breath). UTE scan parameters are as follows: TR/TE = 2.5/0.11 ms, slab-selective excitation with a Shinner-Le Roux minimum phase pulse with time bandwidth product (TBP) = 9 and pulse width (pw) = 0.4 ms [32], flip angle (FA) = 4°, bandwidth (BW) = 125 kHz, FOV 300x300 mm^2^ in the axial plane and 248−274 mm in the superior-inferior dimension depending on size of the lung, 1.1x1.1x1.4 mm^3^ resolution, and no cardiac gating. TE was minimized by matching the end of the minimum phase pulse and the end of the slab-selective gradient ramp down with the VERSE algorithm [33]. Slab rephasing gradient was eliminated such that the center point of radial sampling was shifted from the k-space center along the slab-selective/z axis by approximately 1.5 points. To avoid signal intensity fluctuation at the beginning of data acquisition because of digital filtering, a 20-μs delay was inserted between the onsets of signal reception and readout gradient ramp, which is critical for retrospective gating with k-space point navigator (explained later in detail). Fraction of time to acquire data for pulmonary imaging was 37.3% of the total scan time. To confirm sound-guided respiration during the UTE scan, respiratory physiological signals were monitored with a respiratory bellows and recorded for two subjects.

For comparison, free-breathing UTE scans without the respiration guide were acquired with identical sequence parameters to those in the sound-guided UTE scan. In the free-breathing scans, 111,616 views were continuously acquired, which resulted in scan time of 4:42 including time for dummy scans. 3D k-space was sparsely sampled in every 256 views (640 ms) for implementation of retrospective image-based self-gating; thereby, 436 sparse 3D k-space datasets were acquired. During the free-breathing scan, the subject was instructed to stay relax and freely breathe. In MRI exams, a free-breathing UTE scan was conducted after a 3-plane localizer. Then, a sound-guided UTE scan was performed following a short practice scan of sound-guided respiration.

### Image reconstruction

To investigate stability of breath-by-breath diaphragm positions during sound-guided respiration, data from the sound-guided UTE scan were reconstructed to a 3D image series (one image per breath). Raw k-space data acquired with 20–30 receive channels depending on size of the subject’s lung were compressed to 16 channels by coil compression [34, 35]. Then, the compressed data were reconstructed to a 3D image series, *I*_*t*_, by iteratively minimizing the following equation:

$$I_{t}=\arg\;\underset{x}{\min}\left\{\frac{1}{2}{\left|s-Ex\right|}^{2}+\lambda_{s}{TV}_{s}(x)+\lambda_{t}{TV}_{t}(x)\right\},$$ [1]

where *s* is k-space data from the UTE scan, *E* is the encoding matrix and *TV*_*s*,*t*_ and *λ*_*s*,*t*_ are total variation (TV) operator and regularization parameter in spatial or time dimension, respectively. To accurately detect diaphragmatic motion, the image was reconstructed with higher spatial resolution along the superior-inferior axis (1.8 mm) than that in the axial plane (4.5 mm).

In high spatial resolution reconstruction, a 3D image, *I*, was reconstructed with data from all 46 breaths by solving the following equation:

$$I=\arg\;\underset{x}{\min}\left\{\frac{1}{2}{\left|s-Ex\right|}^{2}+\lambda_{s}{TV}_{s}(x)\right\}.$$ [2]

Data for high-resolution reconstruction were acquired with a relatively high undersampling factor of R ~ 6 (as compared to the Nyquist sampling) at the lowest sampling density area in the radially sampled k-space. Therefore, the high-resolution image was iteratively reconstructed with TV regularization to take advantage of the receive coil sensitivity and the sparsity in TV domain. The final reconstructed image was zero-filled to 0.7 mm isotropic resolution. All image reconstructions in this article were performed with a reconstruction routine written with C++/CUDA.

#### Retrospective gating for free-breathing UTE data

Data from free-breathing UTE scans were reconstructed without gating (w/o gating) and with two retrospective respiratory motion managements, k-space point navigator (k-point navigator) and image-based self-gating (Img-SG). In the w/o gating reconstruction, a high-resolution image was reconstructed from all 111,616 radial views from the free-breathing UTE scan without respiratory motion correction by solving Eq [2].

In the k-point navigator reconstruction, similarly to the k-space center navigator [19, 20], a k-space point at the center of the radial sampling trajectory, which was acquired in every TR period in UTE, was used to track respiratory motion. However, the center point is slightly shifted from the k-space center (by ~1.5 pixels), since pulsed gradients for rephasing the slab-selective gradient were eliminated in this study. Therefore, we refer to this navigator as “k-space point”, not “k-space center”. The shift from the k-space center resulted in a linear phase of 3π over FOV along the superior-inferior axis. The navigator signals were extracted from raw data before coil compression. Since sparse 3D k-space was sampled in every 640 ms (1.56 Hz), low-pass filtering with a raised-cosine filter with a ±1.56 Hz passband and a roll-off factor of 0.33 was applied to a time series of the navigator signal. The low-pass filtering removed intensity fluctuation associated with eddy currents from radially changing readout gradient directions from one TR period to the next [36, 37]. After the low-pass filtering, intensity changes of the navigator signal were mostly related to respiratory motion. Then, a single receive channel with largest standard deviation of the navigator signal was selected for retrospective gating.

Trigger timings for image reconstruction were set by picking up local minima in magnitude of the selected navigator signal. Based on the trigger timings, data were binned to 10 respiration phases. Fluctuation of respiration rate during the MR scan was addressed by evenly stretching each respiration cycle; thereby, each of the 10 respiration phases contained the same number of views. The binned data was first reconstructed to a 3D image series with 10 respiration phases by solving Eq [1], in which spatial resolution of the reconstructed image was 2.8 mm isotropic. Data from 4 exhalation phases with stable diaphragm positions were extracted for high-resolution reconstruction and reconstructed to an image by minimizing Eq [2]. The number of views used for high-resolution reconstruction was 44,144 ± 200 views depending on the first and last trigger timing in the navigator signal. Then, fraction of time for contributing the high-resolution reconstruction was 39.5% on average.

In the Img-SG reconstruction, the free-breathing scan data were reconstructed to a 3D image series with 436 temporal frames (256 views/frame) by solving Eq [1]. Spatial resolution of the time series image was set to 6.7x6.7x2.3 mm^3^, where the resolution is higher along the superior-inferior axis to capture diaphragm motion. Diaphragm position was detected by tracking the lung-liver interface at the top flat region of the right hemidiaphragm. Based on the obtained diaphragm positions, data for high-resolution reconstruction was extracted by picking up frames in the highest 40% diaphragm positions in exhalation (44,544 views) (such that fraction of time for high-resolution reconstruction was 40%). A high-resolution image was reconstructed by minimizing Eq [2]. High-resolution reconstruction data for the three respiratory motion managements (sound-guided respiration, k-point navigator and Img-SG) were acquired with the identical UTE sequence parameters and a comparable number of radial views (41k-44k views) for fair comparison. Image reconstruction parameters were also identical in all the high-resolution reconstructions.

### Evaluation of sharpness of lung-liver interface

Performance of the four respiratory motion managements (sound-guided respiration, w/o gating, k-point navigator and Img-SG) was quantified by measuring a rise distance of the lung-liver interface at the top flat region of the right hemidiaphragm. To quantify the rise distance, intensity profile of the lung-liver interface along the superior-inferior axis was fitted with a sigmoid curve, *v*, [38] given by:

$$v=\frac{a}{1+exp\{b∙(z-z_{0})\}}+v_{0},$$ [3]

where *z* is spatial location along the superior-inferior/z axis and *a*, *b*, *z*_0_ and *v*_0_ are free parameters to be estimated by least square fitting. From the fitted sigmoid curve (Eq [3]), a 10–90% rise distance, *w*_10-90_, was given by:

$$w_{10-90}={z|}_{v=0.1a+v_{0}}-{z|}_{v=0.9a+v_{0}}=\frac{2\;\log\;9}{b}.$$ [4]

### Quantification of blood inflow effects

While RF pulses are constantly applied in the free-breathing UTE scan, the sound-guided scan had long delay time (3.76 sec) before every data acquisition window. To evaluate blood inflow effects during the long delay time, SNR in the lung tissues was measured in the images for sound-guided respiration, k-point navigator and Img-SG. SNR was calculated by *s_lung_*/*σ_noise_*, where *s_lung_* is an mean signal intensity in a region of interest (ROI) selected in the middle of the right lung (3.5x3.5x3.5 = 42.9 cm^3^ volume) and *σ_noise_* is a noise standard deviation. The selected ROI contained blood vessels and lung tissues. *σ_noise_* was calculated from background noise assuming the noise distribution is Gaussian. The standard deviation of the Gaussian distribution (*σ_noise_*) was estimated by fitting the background noise distribution in the magnitude image with the Rayleigh distribution.

To evaluate the blood inflow effects on pulmonary vasculature visualization, visually perceived sharpness was also quantified by calculating an average of acutance, $\bar{A}$, which is given by:

$$\bar{A}=\frac{1}{N_{ROI}}\sum_{r\in ROI}\left|\nabla I(r)\right|,$$ [5]

where *N*_*ROI*_ is the number of pixels in the selected ROI and *I*(***r***) is the high-resolution image reconstructed with Eq [2]. The ROI for acutance calculation was identical to the one for SNR calculation.

### Statistical analysis

Quantitative values in this article were presented as mean ± standard deviation. Differences in means of the four respiration management groups were compared using one-way ANOVA with post-hoc paired t-test in case the ANOVA test indicated significant differences among the group means. *p*-values were adjusted with the Bonferroni correction for multiple comparison. A *p*-value < 0.05 for a two-tailed test was considered to be statistically significant. All statistical tests in this article were performed with the R statistical package [39].

### Renal angiography

The proposed sound-guided respiration was implemented in non-contrast enhanced renal angiography with stack-of-stars balanced steady-state free precession (SoS-bSSFP) sequence with inversion recovery preparation for suppressing stationary background and venous signals (Fig 1B). The sound-guided respiration was set to be identical to that in pulmonary imaging: 6 sec/breath with 1.8 sec for inhalation and the remaining 4.2 sec for exhalation.

A thick inversion RF band that extended 2 cm above to 18 cm below an imaging volume covering both of the left and right kidneys was applied after 0.8 sec from the beginning of the breathe-out instruction. Following previous non-contrast enhanced renal angiography studies, recovery time, TI, was set to 1300 ms to allow sufficient inflow of fresh arterial blood while suppressing background renal tissue signals [40–42]. After the recovery delay, segmented SoS-bSSFP readout started following chemical shift-selective inversion for fat suppression. SoS-bSSFP sequence parameters were as follows: TR/TE = 3.86/1.93 ms, FA = 60°, sinc pulse excitation with pw = 0.8 ms and TBP = 5, BW = 200 kHz, 150 radial views/breath, FOV = 384x384x104 mm^3^, 1.5x1.5x2 mm^3^ resolution, and no cardiac gating. 3D cylindrical k-space was sampled during 35 breaths (5,250 views in total), resulting in scan time of 3:30. Ten bSSFP dummy scans were applied with linearly increasing flip angles to readily reach the steady-state magnetization before starting data acquisition [43] so that *N*_*seg*_ = 160 views. Image reconstruction was performed by solving Eq [2] and the reconstructed image was 2x zero-filled to 0.75x0.75x1 mm^3^ resolution.

To evaluate contrast between arterial blood and background renal tissues, three ROIs were set along the superior-inferior axis in each of the left and right kidney for cortex and medulla. In renal images, signal intensity variations were most conspicuous along the superior-inferior axis mainly due to the B_1_^+/-^ field variation and the excitation profile in SoS-bSSFP. Size of each ROI was set to 3.75x3.75x5 mm^3^; total volume of the 6 ROIs was 421.9 mm^3^ for each of cortex and medulla. Signal intensities of the renal tissues were normalized with arterial blood signal by setting one ROI with a size of 6x6x8 mm^3^ in the aorta where renal arteries bifurcate. Normalized contrast, *C*_*norm*_, was calculated by (*S_blood_*−*S_tissue_*)/*S_blood_*, where *S*_*blood*_ and *S*_*tissue*_ represents the mean arterial blood signal and the mean cortex or medulla signal in the selected ROIs, respectively.

## Results

### Gradient sound respiration guide

**Fig 2:**
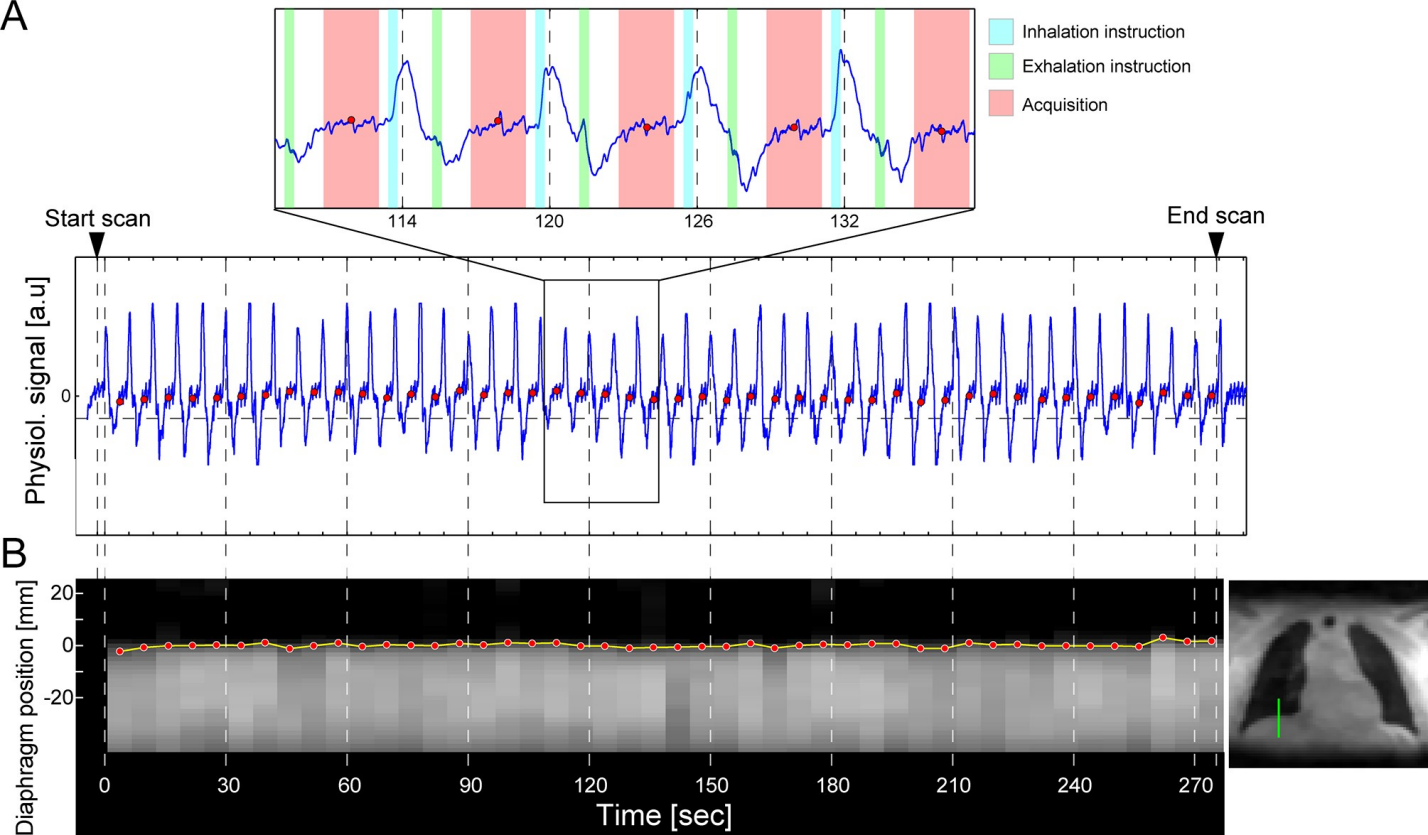
Validation of sound respiration guide. A) Physiological signal recorded with a respiratory bellows during a sound-guided UTE MRI scan shows a regular 6-second period pattern (blue line; lower panel), where sharp peaks represent the onset timings of inhalation. The physiological signal started rising up when the breathe-in instructions (right blue area; upper panel) were turned on and physiological signal drops were detected following the breathe-out instructions (right green area). Data acquisition (red area) was performed during the latter half of the exhalation period right before the subsequent breath-in instruction. Red points represent the center of the UTE acquisition periods. B) Diaphragm positions obtained from breath-by-breath 3D image series reconstruction of the sound-guided UTE MRI scan. Diaphragm position tracked with the lung-liver interface at the top flat region of the right hemidiaphragm (green line in the right image) stayed at a similar position in every breath.

None of subjects reported any discomfort or inconvenience with the sound-guided respiration in pulmonary or renal MRI scans. Physiological signals measured with a respiration bellows during the sound-guided UTE scan showed a regular pattern with a six-second period (Fig 2A). A quick increase of the physiological signal was observed right after onset of the breathe-in instruction, whereas its drop was seen after the breathe-out instruction. The sound-guided respiration facilitated a similar/consistent diaphragm position in every breath by guiding breathing with a constant breathing rate (Fig 2B).

### Retrospective gating for free-breathing UTE data

**Fig 3:**
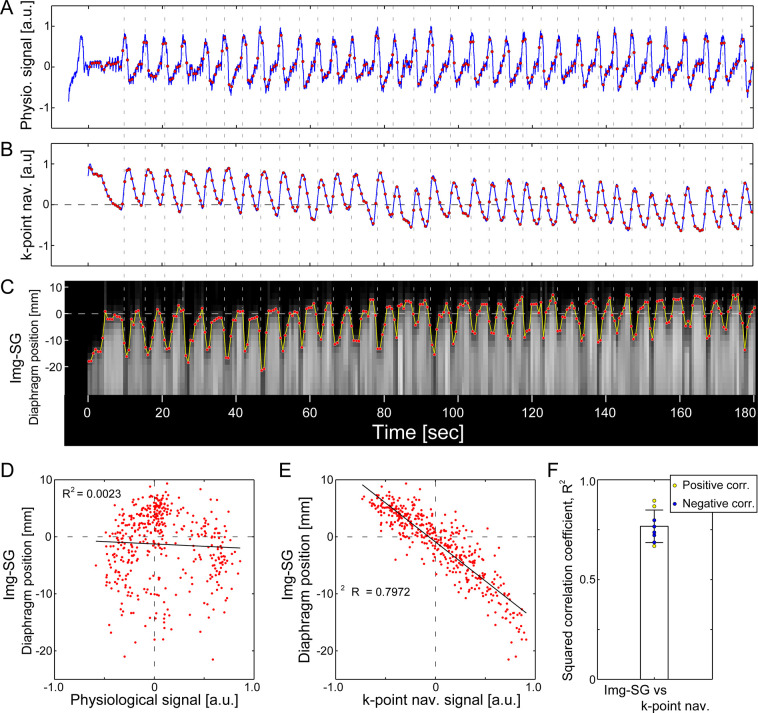
Retrospective gating for a free-breathing UTE MRI scan. A) Physiological signal recorded with a respiratory bellows. B) k-point navigator signal extracted from the center of the radial k-space sampling trajectory. C) Diaphragm position obtained with image-based self-gating (Img-SG). Both k-point navigator and Img-SG captured respiratory motion well; physiological signal peaks matched the ascending slopes of the k-point navigator signal and the timings when the diaphragm started moving down in Img-SG (vertical dotted lines). Red points in A-C represent the timing of 3D low-resolution image acquisition in Img-SG. While the three signals captured respiratory motion, correlation between the physiological signal and the diaphragm position from Img-SG was poor (R^2^ = 0.0023; D). However, the k-point navigator signal and the diaphragm position show relatively strong (negative) correlation (R^2^ = 0.7972; E). The strong correlation between k-point navigator and Img-SG was consistently seen in all eight subjects (R^2^ = 0.7673 ± 0.082; F), but the correlation was positive (yellow points) or negative (blue points) depending on subjects. The data shown in A-E are from the same subject as the one in Fig 2.

For the free-breathing UTE data, two types of retrospective gating strategies, k-point navigator and Img-SG, were applied for respiratory motion management. From a comparison with physiological signal, both the k-point navigator and Img-SG captured respiratory motion; the respiratory signals for one representative subject are shown in Fig 3A–3C. Sharp peaks in the physiological signal, which represented the onset of inhalation, matched the ascending slopes of the k-point navigator signal and the timings that the diaphragm position started moving down for Img-SG. While the physiological signal captured respiration well, it showed a poor correlation with the diaphragm position from Img-SG (R^2^ = 0.0023; Fig 3D). In contrast, the k-point navigator signal was correlated well with the diaphragm position (R^2^ = 0.7972; Fig 3E); there was a gradual decrease of the k-point navigator signal from the beginning to the end of the MRI scan observed, whereas Img-SG showed a similar gradual increase of the diaphragm position (Fig 3B and 3C). Similar correlation between the k-point navigator signal and the diaphragm position tracked with Img-SG was observed in all subjects (R^2^ = 0.7673 ± 0.082, *N* = 8; Fig 3F), where positive and negative correlation was seen in three and five subjects, respectively. Average respiration rate during the free-breathing scan, which was calculated from the trigger timings of the k-point navigator (local minima), was 13.97 ± 3.39 breaths/min (4.54 ± 1.20 sec/breath), which is higher than the respiration rate in sound-guided scans (10 breaths/min).

### Sharpness of lung-liver interface

**Fig 4:**
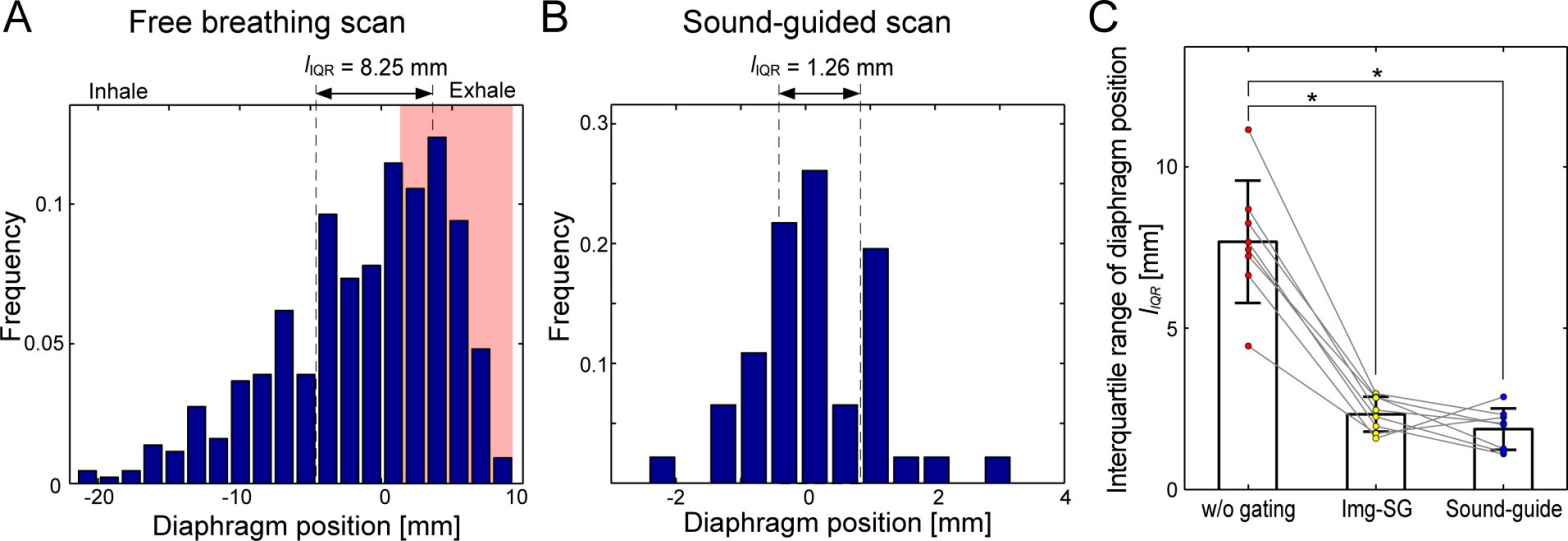
Frequency histogram of the diaphragm positions during free-breathing and sound-guided UTE MRI scans. A) Interquartile range of the diaphragm positions under free-breathing, *l*_*IQR*_, was 8.25 mm. By extracting data at the highest 40% diaphragm positions for image-based self-gating (Img-SG; red area), *l*_IQR_ was reduced to 2.87 mm. B) *l*_*IQR*_ during the sound-guided scan (Sound-guide) was 1.26 mm, which indicates the sound-guided respiration facilitated a similar diaphragm position in every breath. C) The reduction of *l*_*IQR*_ with Img-SG (2.34 ± 0.54 mm) and Sound-guide (1.88 ± 0.64 mm) as compared to free-breathing (w/o gating, *l*_*IQR*_ = 7.69 ± 1.90 mm) was statistically significant (*p* = 0.0003 and 0.0005 for Img-SG and Sound-guide, respectively). The difference of *l*_*IQR*_ between Img-SG and Sound-guide was not significant (*p* = 0.68). * represents *p* < 0.05/3 = 0.0167 with the Bonferroni correction for multiple comparison. Colored points represents *l*_*IQR*_ for individual subjects (*N* = 8). The data shown in A and B is from the same subject as the one in Figs 2 and 3.

Data with the highest 40% diaphragm positions in the free-breathing dataset were selected for high-resolution reconstruction with Img-SG (Fig 4). Selecting the highest 40% diaphragm positions reduced interquartile range (IQR) of the frequency histogram of the diaphragm positions, *l*_*IQR*_; *l*_*IQR*_ decreased from 8.25 mm to 2.87 mm for one representative subject (Fig 4A). Sound-guided respiration provided a comparable *l*_*IQR*_ (= 1.26 mm) to the highest 40% diaphragm positions in free breathing (Fig 4B). The improvement of *l*_*IQR*_ with Img-SG (2.34 ± 0.54 mm) and sound-guided respiration (1.88 ± 0.64 mm) with respect to *l*_*IQR*_ of all diaphragm positions during the free-breathing scan (7.69 ± 1.90 mm) was statistically significant (*p* = 0.0003 and 0.0005 for Img-SG and sound-guided respiration, respectively; Fig 4C). *l*_*IQR*_ for sound-guided respiration was smaller than that for Img-SG, but the difference was not significant (*p* = 0.68).

**Fig 5:**
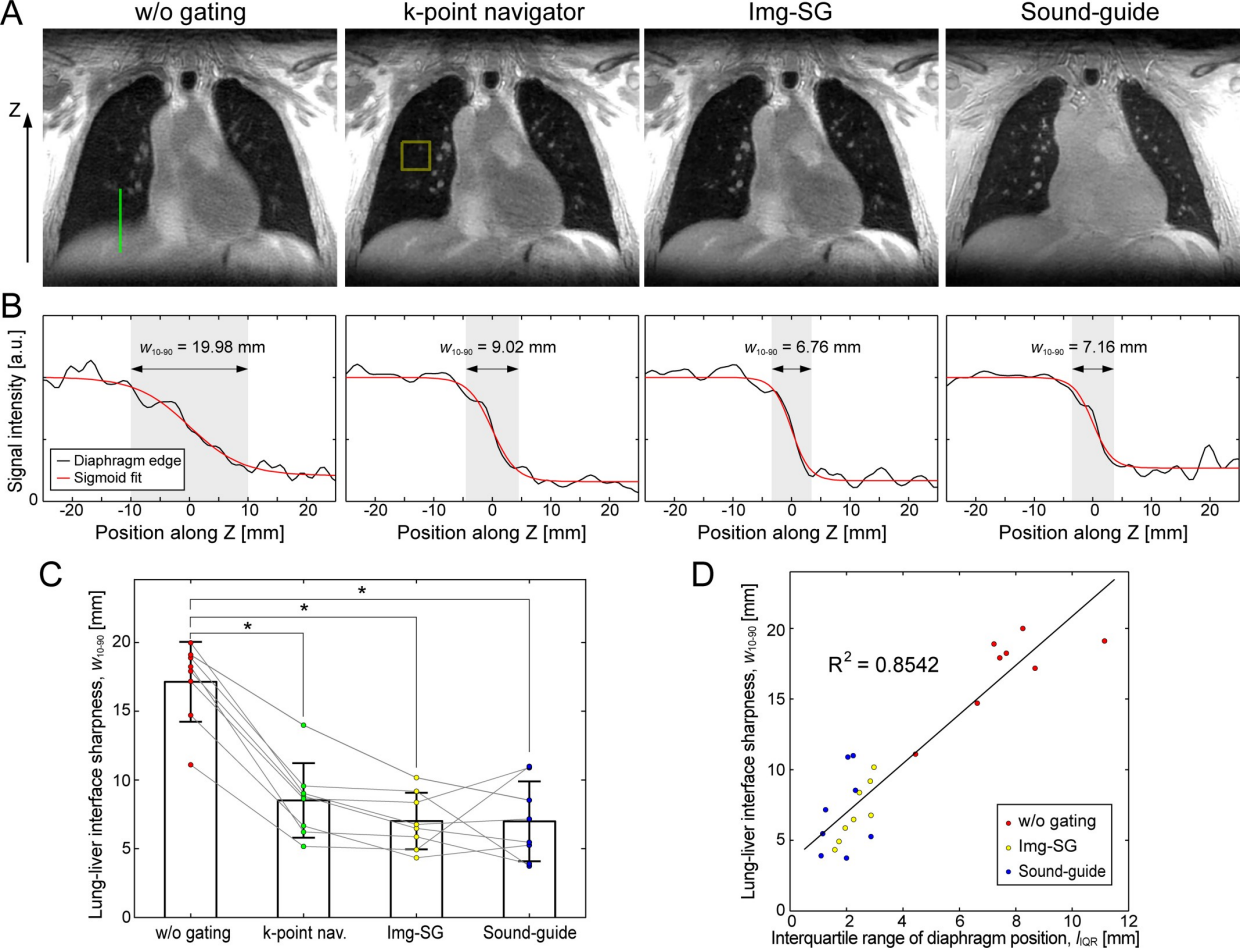
Sharpness of the lung-liver interface for high-resolution UTE MR images. A) Images from k-point navigator, image-based self-gating (Img-SG) and sound-guided respiration (Sound-guide) show visually clear improvement in the lung-liver interface as compared to free-breathing without gating (w/o gating). B) The sharpness of the lung-liver interface was quantified by fitting the intensity profile at the top flat region in the right hemidiaphragm (green line in A) with a sigmoid function. C) Reduction of the 10–90% rise distance, *w*_10-90_, with k-point navigator (8.51 ± 2.71 mm), Img-SG (7.01 ± 2.06 mm) and Sound-guide (6.99 ± 2.90 mm) relative to w/o gating (17.13 ± 2.91 mm) was statistically significant (*p* < 0.0001 for all of k-point navigator, Img-SG and Sound-guide). *w*_10-90_ for Img-SG and Sound-guide was narrower than k-point navigator, but not statistically significant (*p* = 0.017 and 0.31 for Img-SG and Sound-guide, respectively). D) The sharpness of the lung-liver interface in the high-resolution images was linearly correlated with interquartile range of the diaphragm position *l*_*IQR*_ shown in Fig 4C (R^2^ = 0.8542). Colored points represent the values for individual subjects (*N* = 8). * represents *p* < 0.05/6 = 0.0083 in C with the Bonferroni adjustment for multiple comparison. The data shown in A and B are from the same subject as the one in Figs 2–4.

High-resolution lung images reconstructed from the sound-guided UTE scan or the free-breathing UTE scan with retrospective gating (k-point navigator or Img-SG) show visually clear improvement of the lung-liver interfaces as compared to that in an image reconstructed without respiratory gating (w/o gating; Fig 5A). Sharpness of the lung-liver interfaces was quantified with the least square fitting with a sigmoid curve (Fig 5B). A 10–90% rise distance, *w*_*10-90*_, in the image reconstructed with respiratory motion managements (k-point navigator, 8.51 ± 2.71 mm; Img-SG, 7.01 ± 2.06 mm; sound-guided respiration, 6.99 ± 2.90 mm) was significantly smaller than that without gating (17.13 ± 2.91 mm; *p* < 0.0001 for all of k-point navigator, Img-SG and sound-guided respiration; Fig 5C). The lung-liver interfaces in the images from Img-SG and sound-guide respiration were sharper than that from k-point navigator, but the differences were not statistically significant with the Bonferroni adjustment for multiple comparison (*p* = 0.017 and 0.31 for Img-SG and sound-guided respiration, respectively). *w*_*10-90*_ for Img-SG was comparable to that for sound-guided respiration (*p* = 0.99). The sharpness of the lung-liver interface was linearly correlated well with the interquartile range of the diaphragm positions in the free-breathing or sound-guided scan data for high-resolution reconstruction (R^2^ = 0.8542; Fig 5D).

### Pulmonary angiography

**Fig 6:**
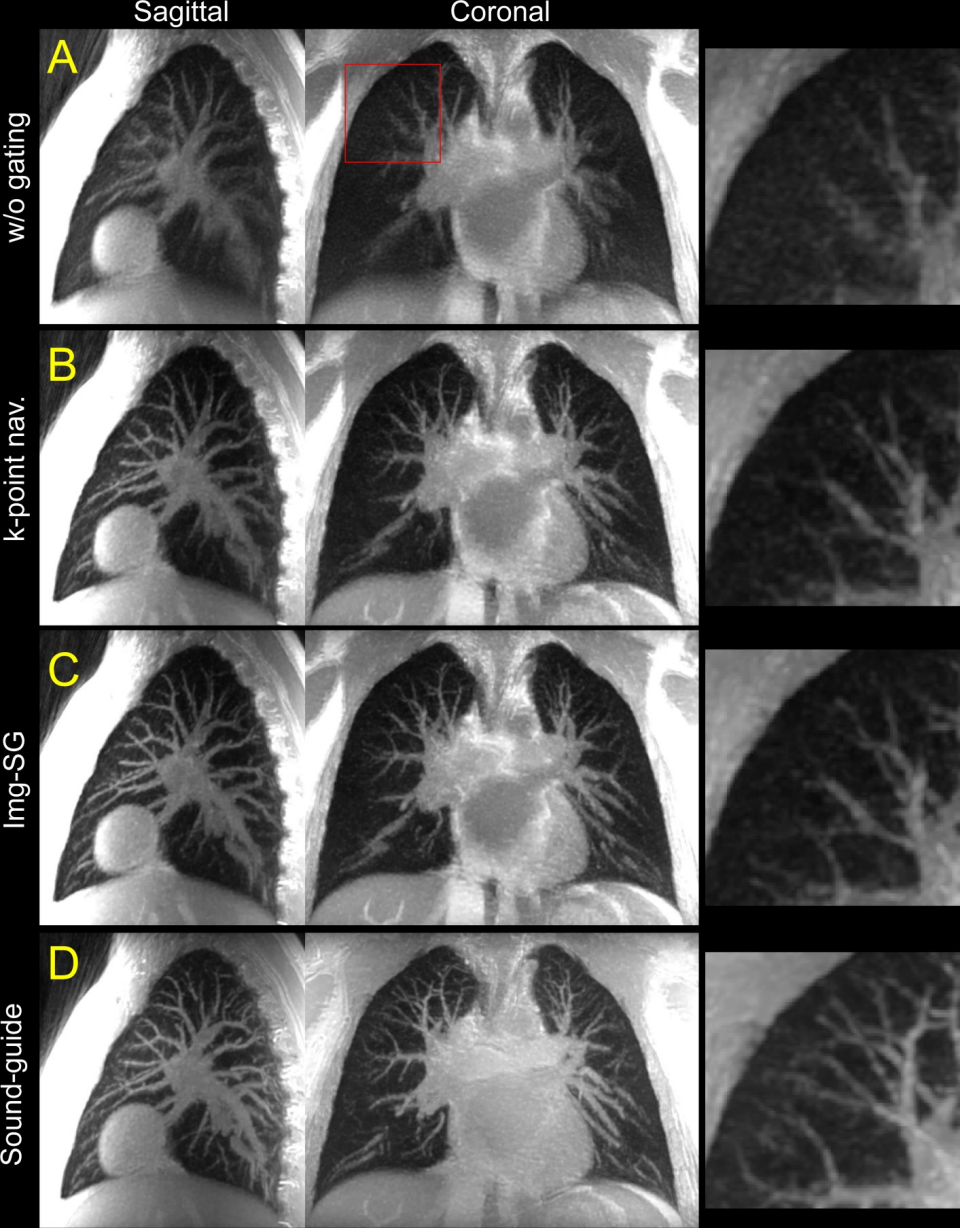
Maximum intensity projection (MIP) of high-resolution UTE MRI images. MIP images for free-breathing without respiratory motion management (w/o gating, A) and free-breathing with retrospective respiratory gating (k-point navigator, B, or image-based self-gating [Img-SG], C), and sound-guided respiration (Sound-guide, D) are presented in sagittal (left) and coronal (center) views. Images obtained with respiratory motion management (B-D) clearly show improved delineation of pulmonary vasculature as compared to w/o gating (A). Moreover, the MIP image from Sound-guide shows notably better visualization for peripheral blood vessels in zoomed views as compared to k-point navigator and Img-SG (right). The MIP images were computed from a 2.1-cm thick slab for both sagittal and coronal views. The images shown in this figure are from the same subject as the one in Figs 2–5. Coronal MIP images with a projection slab sliding from posterior to anterior are shown in S1 Video.

UTE lung images inherently show strong contrasts between blood vessels and lung tissues such that pulmonary vasculature was well-visualized without exogenous contrast mechanism. While radial sampling in UTE is relatively tolerant to motion artifacts, considerable image blurring in pulmonary vasculature was seen in images reconstructed without respiratory motion management particularly in regions around the diaphragm in MIP images (Fig 6A). Such image blurring was noticeably improved by applying retrospective gating (k-point navigator or Img-SG) or sound-guided respiration (Fig 6B–6D). The MIP images from the sound-guided UTE scans more clearly visualized small blood vessels as compared to those from retrospective respiratory gating (k-point navigator or Img-SG). Such improvement of the vasculature delineation was commonly observed in all eight subjects; however, three subjects showed less conspicuous improvement (S1 Fig).

### Quantification of blood inflow effects

**Fig 7:**
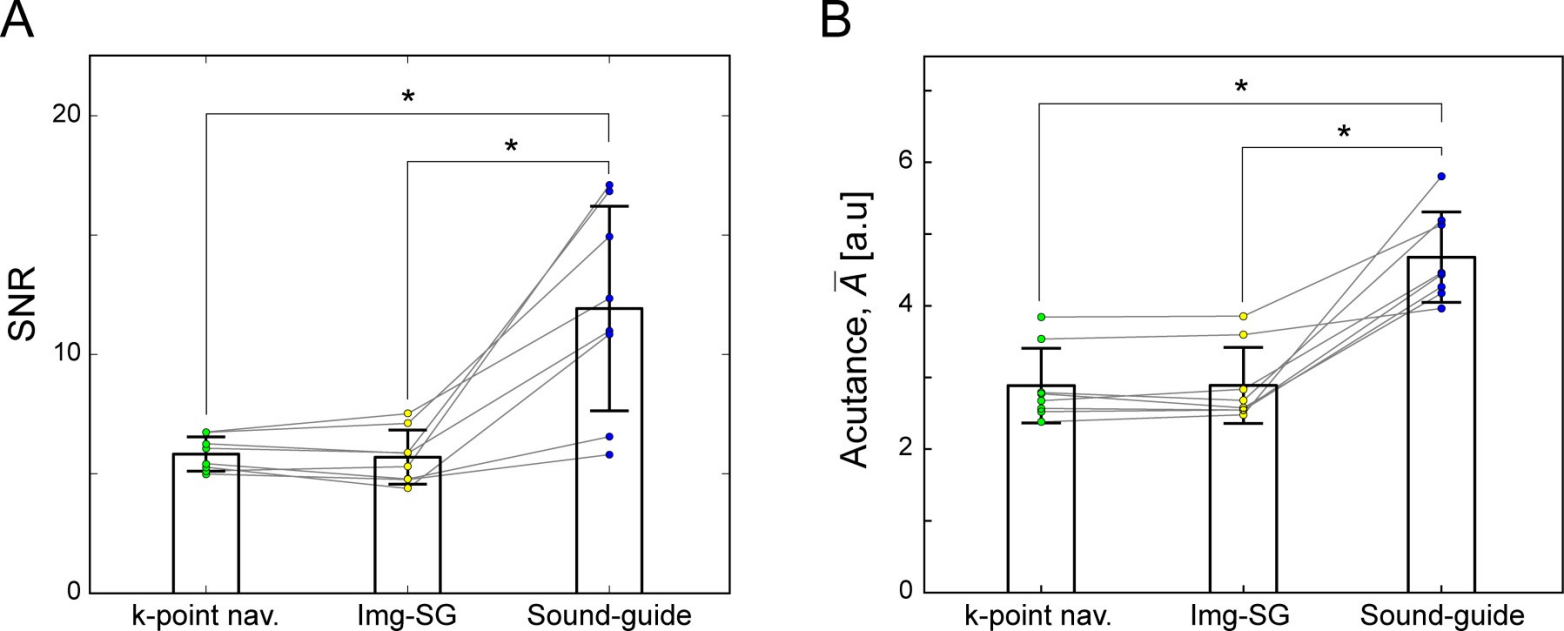
Quantification of blood inflow effects. A) Signal-to-noise ratio (SNR) of the lung tissues was significantly higher with Sound-guide than with k-point navigator (*p* = 0.0038) and Img-SG (*p* = 0.0027), but the difference between k-point navigator and Img-SG was not significant (*p* = 0.52). A cubic ROI selected in the right lung is shown in the k-point navigator image in Fig 5A with a yellow box. B) An average of acutance, $\bar{A}$, was calculated for the identical ROI to SNR calculation. The $\bar{A}$ values were significantly larger for sound-guided respiration than for k-point navigator (*p* = 0.0007) and Img-SG (*p* = 0.0006). The difference between k-point navigator and Img-SG was not significant (*p* = 0.94). Colored points represent the values for individual subjects (*N* = 8). * represents *p* < 0.05/3 = 0.017 with Bonferroni adjustment for multiple comparison.

To evaluate effects of arterial blood inflow during the long delay time between acquisition windows in sound-guided respiration UTE, SNR in the lung tissues was compared for the three respiratory motion managements (SNR = 5.83±0.72, 5.70±1.13 and 11.93±4.29 for k-point navigator, Img-SG and sound-guided respiration, respectively; Fig 7A). The SNR in the sound-guided respiration UTE images was significantly higher than that for k-point navigator (*p* = 0.0038) and Img-SG (*p* = 0.0027). The difference between k-point navigator and Img-SG was not significant (*p* = 0.52). Visually perceived sharpness of pulmonary vasculature visualization was quantified for the UTE images with sound-guided respiration ($\bar{A}=4.68\pm 0.63$), k-point navigator ($\bar{A}=2.89\pm 0.52$) and Img-SG ($\bar{A}=2.89\pm 0.53$). Similarly to SNR, the $\bar{A}$ values for sound-guided respiration were significantly higher than those for k-point navigator (*p* = 0.0007) and Img-SG (*p* = 0.0006) due to an increase of the blood inflow effects. The difference between k-point navigator and Img-SG was not significant (*p* = 0.94).

### Renal angiography

**Fig 8:**
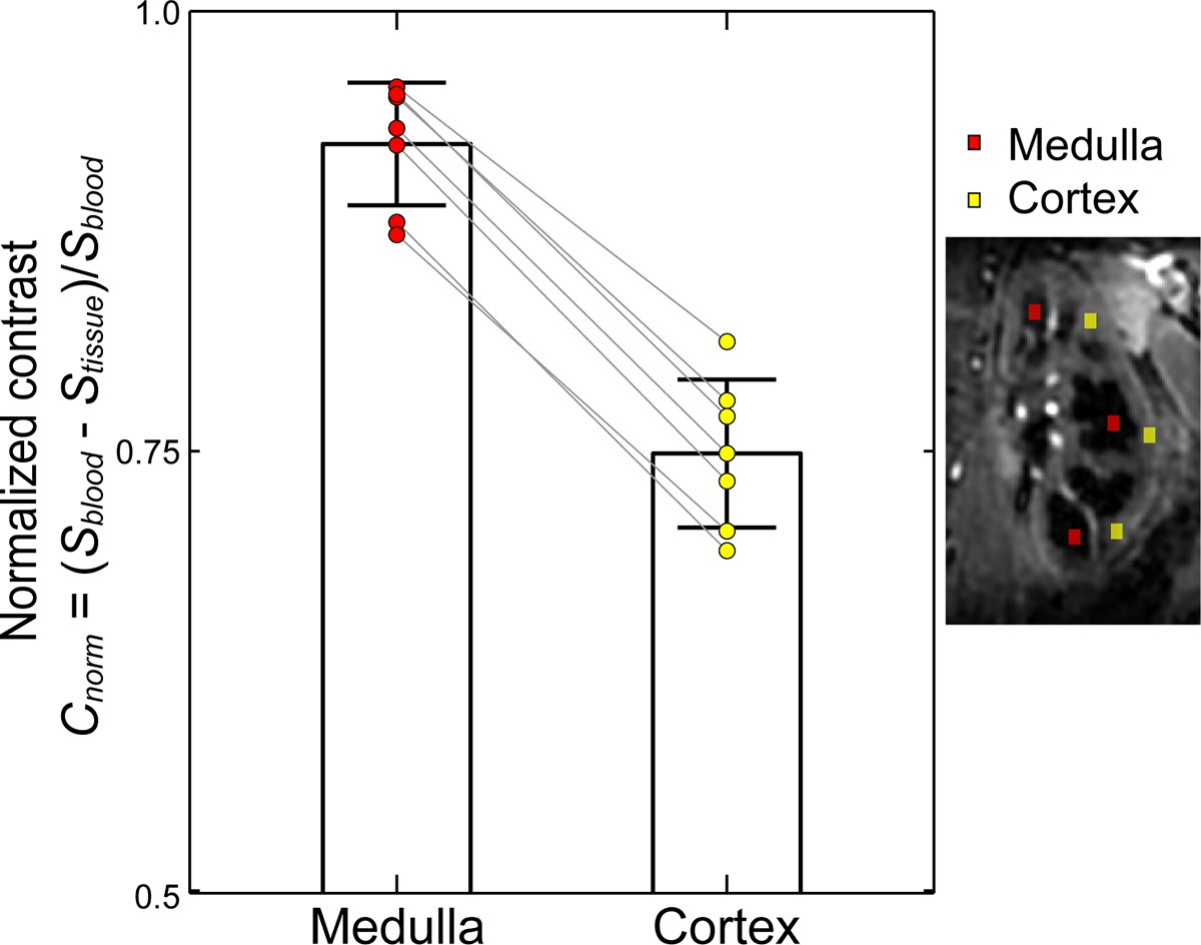
Image contrast between renal tissues (cortex and medulla) and arterial blood. Renal arteries are seen with strong contrasts relative to background renal tissues (right image). Normalized contrast, *C*_*norm*_ = (*S*_*blood*_−*S*_*tissue*_)/*S*_*blood*_, was calculated by setting 3 regions of interests (ROIs) in each of left and right kidneys; representative locations of the ROIs in the left kidney are shown for cortex (yellow) and medulla (red). The normalized contrast was 0.75 ± 0.04 and 0.92 ± 0.03 for cortex and medulla, respectively. Colored points represent the values for individual subjects (*N* = 7). The reference arterial blood signal, *S*_*blood*_, was obtained from a ROI located in the aorta where renal arteries bifurcate.

**Fig 9:**
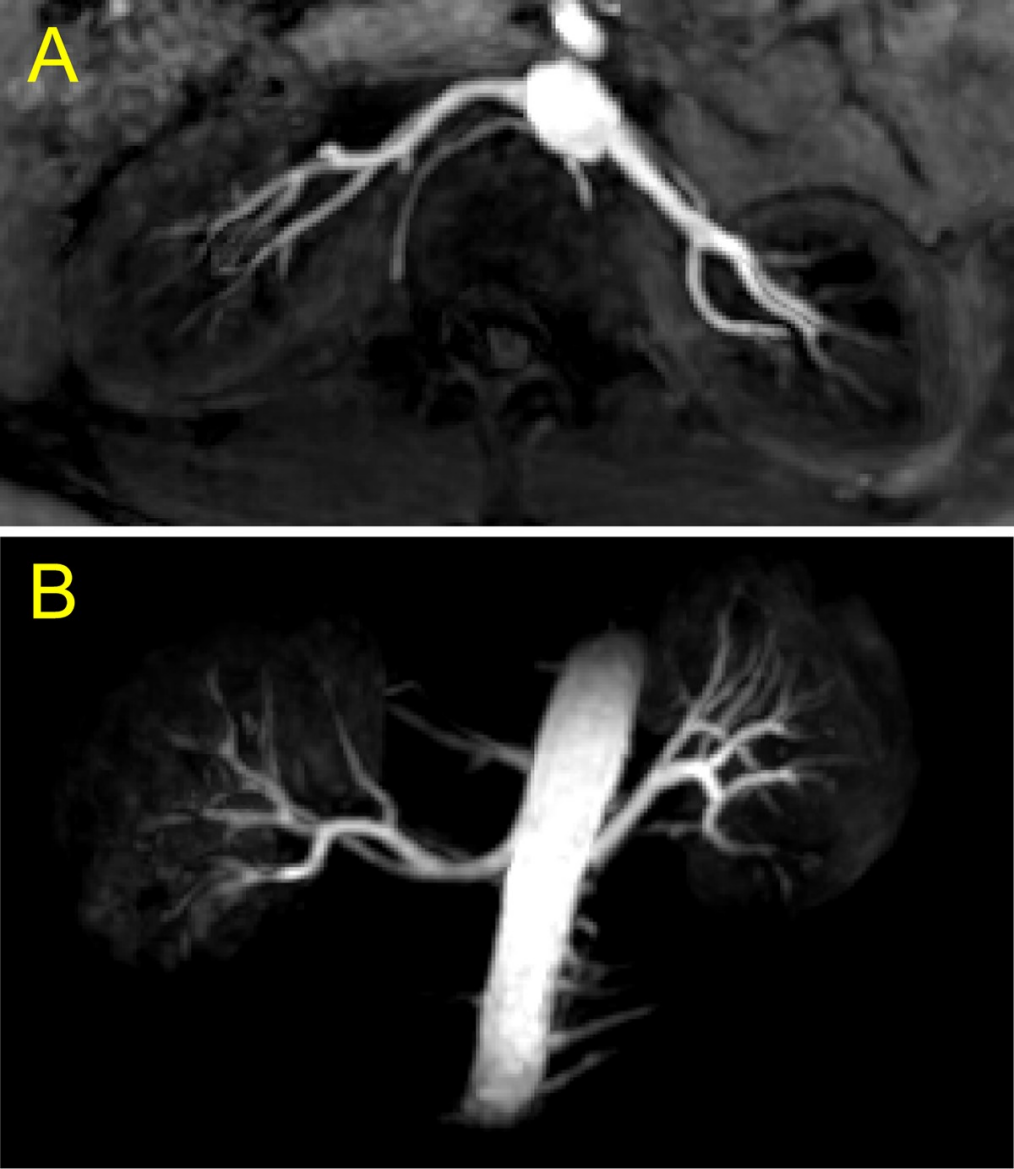
Maximum intensity projection (MIP) of a SoS-bSSFP renal angiography image. Projection was calculated with a 1.5-cm thick slab in axial view (A) and the entire kidneys in coronal view (B). To clearly show renal arteries, surrounding tissues except kidneys and arteries were masked out in B. MIP images projected onto other image planes are shown in S2 Video.

The sound-guided respiration validated in UTE pulmonary imaging was implemented in non-contrast enhanced renal angiography with segmented SoS-bSSFP and slab-selective inversion recovery for background tissue suppression. Background renal tissues were suppressed well so that renal arteries were clearly delineated with strong contrasts in reconstructed images for all seven subjects (*C*_*norm*_ = 0.75 ± 0.04 and 0.92 ± 0.03 for cortex and medulla, respectively; Fig 8). Renal angiography with the sound-guided respiration clearly visualized renal, segmental and interlobar arteries in MIP images (Fig 9).

## Discussion

Respiration guide using acoustic sounds generated by pulsed gradient waveforms has been introduced for compensating respiratory motion during MRI scans. Short instruction of the sound-guided respiration before MRI exams using a sound recording, which typically took less than five minutes, and a 1.5-min practice scan in the MRI scanner successfully facilitated study subjects to breathe with a consistent respiration pattern, which could compensate respiratory motion comparably to retrospective respiratory gating with Img-SG or k-point navigator.

In this study, respiration rate was fixed to 10 breaths/min in all the sound-guided MRI scans, whereas respiration during the free-breathing MRI scans was faster than the sound-guided respiration for most of the subjects in the pulmonary imaging study (7/8). In healthy adults, respiration rate is typically 12–20 breaths/min with free-breathing [44]. Respiration guide with a matched respiration rate with free-breathing may improve stability and comfort of the sound-guided respiration. Adjustment of respiration rate for individual subjects would particularly be important for patients with reduced lung function to comfortably breathe with a consistent respiration pattern during sound-guided MRI scans.

In the sound-guided pulmonary scans, each data acquisition window was approximately 2 seconds in the latter half of the exhalation period, which is longer than the acquisition window for Img-SG in the free-breathing scans (0.64 seconds). It is shown that respiratory motion within the acquisition window is one important cause of motion artifacts [45–47]; the diaphragm position moves up to 2 mm during an acquisition window as short as 150 ms. Although the acquisition window in the sound-guided pulmonary UTE MRI in this study was set in a period of the stable diaphragm position in exhalation, the data acquired during the long acquisition window may see impacts from residual respiratory motion. Adjustment of respiration rate in the sound-guided respiration might help reduce respiratory motion within the acquisition window and stabilize the diaphragm position.

In this study, the subjects were instructed to stay relaxed without intentional breath-holding during the data acquisition period/window in sound-guided scans. Although breath-holding may reduce residual respiratory motion within the acquisition window, multiple breath-hold MRI scans often work poorly because of inconsistent diaphragm positions between breath-holds [8]. Further investigation is required to minimize respiratory motion within the acquisition window and fluctuation of the diaphragm position breath by breath.

One shortcoming of the sound-guided respiration is to require subject compliance to breathe following the instruction sounds. In cases subjects have difficulties to follow the respiration sound instruction (e.g. hard to keep up a consistent breathing pattern and fall into a sleep), the sound-guided respiration may fail to compensate respiratory motion. To reduce deleterious impacts in such cases, the sound-guided respiration can be easily combined with standard respiratory gating methods such as physiological monitoring with a respiration bellows and navigator acquisition. The additional respiratory monitoring/triggering can confirm data acquisition in common respiration phase while guiding a constant/consistent breathing pattern with sound-guided respiration. For the purpose of additional monitoring of respiratory motion, acquiring a 1D projection consistently [48, 49] can be readily incorporated in radial MRI by adjusting the view order without inserting extra navigator acquisition.

Retrospective respiratory gating with k-point navigator and Img-SG was investigated to evaluate performance of the gradient sound guided respiration. The k-point navigator is a similar 0D navigator to the k-space center navigator [5, 14–20], but the navigator point in k-space is slightly shifted from the k-space center, because the slab-selective gradient is not rephased in the UTE sequence herein. This slight shift in k-space domain introduces linear phase in spatial domain along the slab-selective/superior-inferior axis, which is a primary axis of the diaphragm motion. The extra linear phase along the motion axis might help detect respiratory motion from the navigator signal. The k-point navigator signals in this study were well-correlated with the diaphragm positions obtained from Img-SG (Fig 3E and 3F).

The k-point navigator signal showed positive or negative correlation with the diaphragm position. The k-space center signal is also known to correlate with the diaphragm position positively or negatively depending on the relative position of the receive coil and the diaphragm [5, 6]. In this study, a single receive channel was selected for retrospective gating with k-point navigator using an automatic algorithm of picking up one channel with the largest standard deviation of magnitude of the navigator signal. For k-space center navigator, more complex calculation using principal component analysis [15, 26, 50] or coil clustering [6] were introduced to extract the navigator signal from multiple receive channel data. Similar strategies could be applied in respiratory gating with k-point navigator. Besides, it is known that signal changes associated with respiratory motion are observed not only in magnitude of the navigator signal but also in phase [15, 19]. Further investigation is required to improve the respiratory motion tracking with k-point navigator in future studies.

Pulmonary UTE images clearly delineated the pulmonary vasculature with strong image contrasts because of the large proton/tissue density difference between blood and lung tissues and the short echo time that can preserve fast-decaying signals from the small blood vessels in strong susceptibility regions. The UTE images from the sound-guided scans showed better visualization of the peripheral blood vessels as compared to the images from the free-breathing scans. In the sound-guided scans, there is long delay time (approximately 4 seconds) between one data acquisition period to the next, which allows fresh arterial blood to flow into the lung and increases signals from small peripheral blood vessels.

One drawback of using sound-guided respiration is data acquisition of the transient-state magnetization asymptotically approaching the steady-state, since the long delay time from one acquisition window to the next allows significant inflow of fresh arterial blood and T_1_ relaxation in the longitudinal magnetization. In this study, the flip angle used in UTE scans is low (4°) and as such the magnetization slowly reached the steady-state. Therefore, there was no clear artifact associated with the transient-state magnetization in the sound-guided respiration UTE scans. However, such signal fluctuation in the transient-state magnetization will become more serious when using higher flip angles. To reduce the impacts from the transient-state acquisition, one can insert dummy scans at the beginning of each acquisition window. However, dummy scans may reduce strong blood signal intensities associated with the arterial blood inflow. Another solution to the transient-state acquisition is to use variable flip angles in UTE acquisition to make the transient-state magnetization flat, as was introduced in bSSFP [51] and 3D TSE [52] in previous works.

Proton density weighting in pulmonary UTE MRI is advantageous for pulmonary angiography, but it may be hard to differentiate blood vessels and some pathology such as mucoid plugging in cystic fibrosis patients. To solve such ambiguity, the black-blood preparation technique has recently been introduced in pulmonary UTE MRI and differentiated mucoid plugging and blood vessels in cystic fibrosis patients [53]. The sound-guided respiration developed herein can readily be implemented in the black-blood UTE sequence in a similar way to that in renal angiography in this study. Moreover, the sound-guided respiration can also be easily combined with other pulmonary angiography techniques using bSSFP [9] and QISS [54].

The gradient sound guided respiration validated in pulmonary imaging studies was implemented with SoS-bSSFP for non-contrast enhanced renal angiography. The SoS-bSSFP renal angiography with sound-guided respiration visualized renal arteries with strong contrasts between arterial blood and renal tissues. In addition to sound-guided respiration, oversampling of the k-space center region with 2D radial sampling in SoS-bSSFP makes it tolerant to inflow and residual motion artifacts [55]. Furthermore, radial k-space sampling is more immune to undersampling artifacts than Cartesian sampling such that it can increase efficiency of image reconstruction with sparsity constraints (i.e., compressed sensing reconstruction).

Fat suppression is important for many of bSSFP imaging applications such as renal angiography and cardiac coronary imaging. In this study, fat signals were suppressed by applying spectral-selective fat inversion right before segmented SoS-bSSFP readout. With bSSFP, robust fat suppression can be achieved by setting TR to match the fat resonance frequency and the bSSFP stop band, but it is hard to set such TR at 3 T or higher fields, since the bSSFP band spacing gets narrower as compared to the fat chemical shift frequency along with an increase of the field strength. Alternate TR bSSFP also known as wideband bSSFP was previously introduced to reduce banding artifacts in bSSFP by increasing the bSSFP band spacing [56, 57]. By matching the bSSFP stop band and the fat frequency, alternate TR bSSFP achieved more efficient fat suppression at 3T [58]. Use of alternate TR bSSFP may be another choice for fat suppression in non-contrast enhanced renal angiography.

The application of the proposed sound-guided respiration is not limited to pulmonary imaging and renal angiography demonstrated herein. The sound-guided respiration can be applied to any MRI techniques with respiratory gating such as cardiac coronary angiography, thoracic 4D flow imaging and liver imaging. Constant breathing rate guided by the sound-guided respiration may also be advantageous for quantitative measurements such as T_1_ and T_2_ mapping. Constant breathing interval between the trigger timings with sound-guided respiration can form a consistent longitudinal magnetization in every acquisition window. Standard respiratory gating using a respiratory bellows or navigator acquisition for free breathing often see fluctuation of the breathing rate, which results in fluctuating longitudinal magnetization at the trigger timings.

In conclusion, respiration guide using acoustic sounds generated by pulsed gradient waveforms was introduced in MRI. The sound-guided respiration successfully compensated respiratory motion during MRI scans; its performance was comparable to retrospective respiratory gating with k-point navigator or image-based self-gating. Non-contrast enhanced renal angiography with the sound-guided respiration achieved clear visualization of renal arteries with strong contrasts.

## Supporting information

S1 Audio Respiration guide sounds generated by pulsed gradient waveforms. Study subjects heard three types of sounds in every 6-sec period during sound-guided MRI scans. Sinusoidal gradient waveforms with two different frequencies generate high pitch breathe-in instruction (1st sound) and low pitch breathe-out instruction (2nd sound). The 3rd one herein is a sound from pulmonary UTE MRI.
(WAV)

S1 Video Maximum intensity projection (MIP) of high-resolution pulmonary UTE MRI images in coronal view. MIP images for sound-guided respiration more clearly visualized pulmonary vasculature (top left: without gating, top right: k-point navigator, bottom left: image-based self-gating and bottom right: sound-guided respiration). The projection slab is 2.1-cm thick and slides from posterior to anterior.
(MP4)

S2 Video Maximum intensity projection (MIP) of a SoS-bSSFP renal angiography image. The MIP images clearly show renal, segmental and interlobar arteries. The projection plane was rotated around the left-right/x axis.
(MP4)

S1 Fig Maximum intensity projection (MIP) of high-resolution UTE MRI images in sagittal (left) and coronal (center) views. Improvement of pulmonary vasculature delineation for sound-guided respiration (Sound-guide, D) as compared to free-breathing without gating (w/o gating, A) and with retrospective gating (k-point navigator, B, or image-based self-gating [Img-SG], C) is observed for visualization of peripheral blood vessels (arrowheads), but less conspicuous for this subject than the one in Fig 6.
(TIF)

S1 Data Relevant data to the study. (MAT)
