# Supplementary figures and images for: Magnetic resonance imaging with gradient sound respiration guide
Source: PLoS One. 2021 Jul 19;16(7):e0254758. doi: 10.1371/journal.pone.0254758 (PMC8289037; doi:10.1371/journal.pone.0254758)

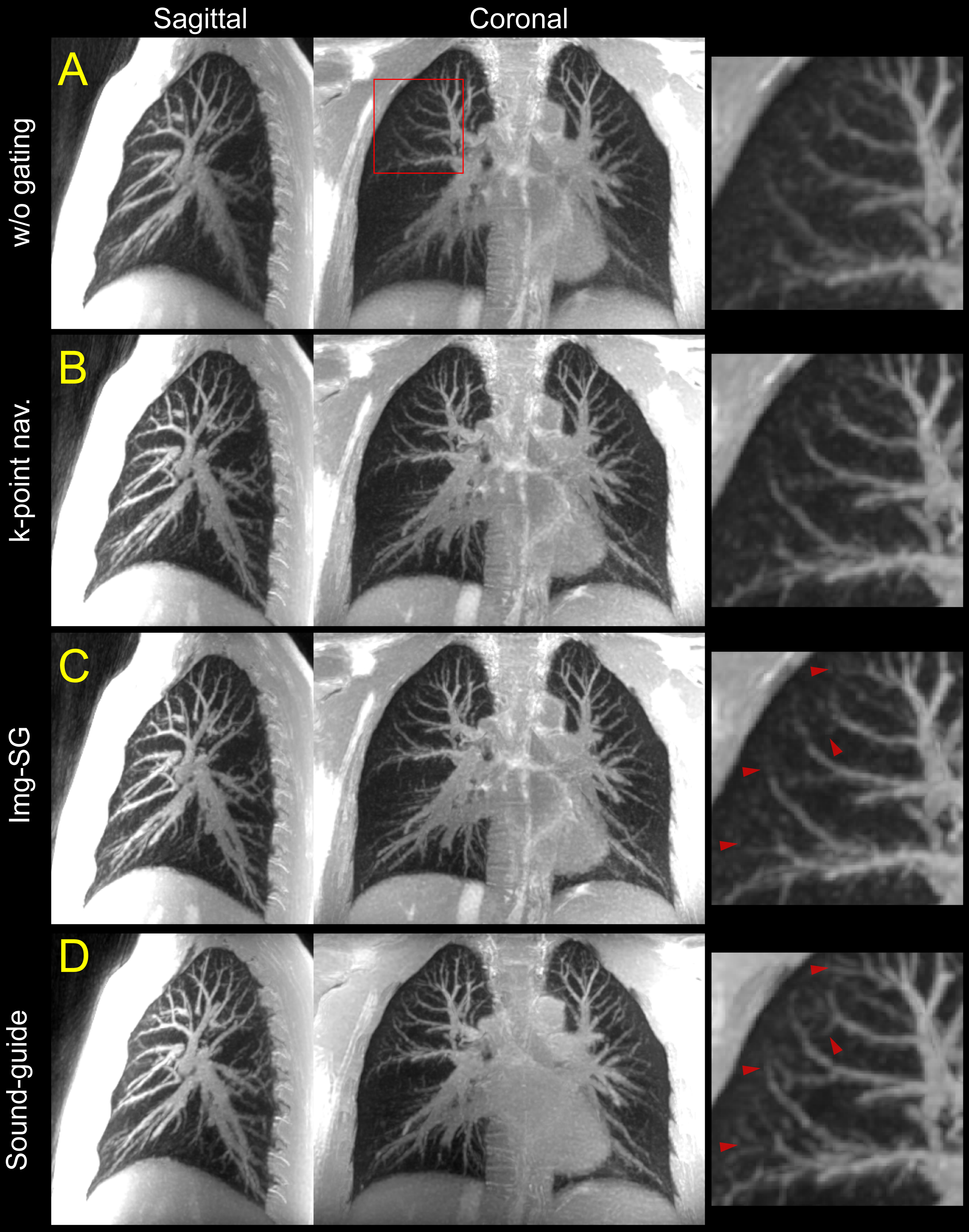

Supplement: S1 Fig — Improvement of pulmonary vasculature delineation for sound-guided respiration (Sound-guide, D) as compared to free-breathing without gating (w/o gating, A) and with retrospective gating (k-point navigator, B, or image-based self-gating [Img-SG], C) is observed for visualization of peripheral blood vessels (arrowheads), but less conspicuous for this subject than the one in Fig 6. (TIF) [file pone.0254758.s004.tif]
